# From ‘government’ to ‘governance’: Tensions in disaster-resilience leadership in Zimbabwe

**DOI:** 10.4102/jamba.v7i1.188

**Published:** 2015-11-30

**Authors:** Pathias P. Bongo, Siambabala B. Manyena

**Affiliations:** 1 Disaster Management and Development Studies, Bindura University of Science Education, Zimbabwe; 2 Disaster Resilience, University of Manchester, United Kingdom

## Abstract

This article examines the challenges that disaster leadership faces to move away from a top-down, command-and-control style to distributed leadership. The article challenges the Sendai Framework for Disaster Risk Reduction which appears to be silent on leadership and instead emphasises ‘good governance’ to enhance organisational and institutional capacity for disaster resilience. We posit that leadership is an indispensable component of good governance, and not emphasising it could be tantamount to a gross underestimation of disaster policy and practice. Using the data from participatory action research that was conducted in Matabeleland South Province, Zimbabwe, the findings reveal some tensions in shifting from command and control to distributed leadership in disaster-risk reduction, which has implications for the shift from government to governance in disaster risks. More importantly, this study reiterates the blurred distinctions between disaster-risk reduction and sustainable development. Thus, unless well-known, sustainable development challenges are addressed – particularly community-based leadership, good governance, the integration of local knowledge, empowerment and ownership of development programmes – shifting from government to disaster governance is likely to continue facing challenges.

## Introduction

The general public expects strong leadership in addressing issues of disaster-risk reduction (DRR) (Kapucu, Arslan & Demiroz 2010; Kusumasari, Alam & Siddiqui 2010). Yet, despite the fact that the literature on disaster-risk reduction has grown over the years, little research has been done on disaster leadership. DRR is used here to mean the concept and practice of reducing the risks of disasters where the leaders influence their stakeholders to employ systematic efforts to analyse and manage the factors causing disasters. These efforts include reducing exposure to hazards, lessening vulnerability of people and property, wise management of land and the environment and improving preparedness for adverse events (UNISDR 2009). Thus, leadership can be viewed as a catalyst ingredient for enhancing resilience or the ability of an individual, community, neighbourhood, institution or system to cope *positively* with rapid-onset shocks or significant and protracted sources of stress (Adger *et al.* 2005; Aldrich 2012; Holling 1973; Norris *et al.* 2008).

Despite the importance of leadership, the global DRR policy, the Sendai Framework for Disaster Risk Reduction (2015–2030) (SFDRR), and its predecessor, the Hyogo Framework for Action 2005–2015 (HFA), appear not to have valued the importance of leadership. Instead, leadership is, perhaps, implied under the rubric of ‘good governance’. Whilst Priority 2 of the SFDRR recognises the importance of strengthening disaster-risk governance to manage disaster risk, leadership is also not directly addressed. We posit that DRR leadership is an indispensable component of good governance, and, as this article demonstrates, negating or paying lip-service to leadership could be tantamount to a gross underestimation of disaster policy and practice. From this vantage point, interrogating DRR leadership could provide a barometer for testing DRR governance.

Thus, this article examines the challenges of disaster leadership within the context of DRR governance in relationship to the distribution of power amongst actors (Lebel *et al.* 2006; Van Asselt & Renn 2011). The article is based on experiences from a participatory-action research (PAR) study performed in Matabeleland South Province, Zimbabwe. The project used PAR, involving training workshops to strengthen DRR organisational capacity and leadership.

The rest of this article is organised into five main sections and begins by discussing the conceptual framework. Here, we examine the notions of leadership more widely, focusing on those that may also be applicable to DRR. This is followed by a reflection on the methodology where we reflect on the role of PAR in initiating social change as well as the ethical issues it raises. We then move to outline Zimbabwe’s DRR framework before presenting the hazard and vulnerability context for Matabeleland South Province, which also serves as the justification of the action-research project. This is followed by a presentation of three cases of DRR governance leadership to exemplify, but also provide a microcosm of, the tensions in DRR governance leadership in Zimbabwe. Finally, a discussion ensues on the leadership attributes articulated in the three cases studies. We conclude that good disaster-governance approaches should be underpinned by DRR leadership frameworks, which should consistently emphasise distributed leadership and power, ownership, coordination, partnership and accountability.

## Disaster governance and disaster leadership

Disaster governance draws from the extensive literature on governance and applies governance principles to risk and risk-reduction domains (Blanco 2015; Fan 2015; Jones *et al.* 2014; Lassa 2010; Tierney 2012; Zurita *et al.* 2015). Van Asselt and Renn (2011) remind us that governance involves a multitude of actors and processes that lead to collectively binding decisions. Lebel *et al.* (2006) view governance as the structures and processes by which communities share power. Thus, governance pays attention to the distribution of political power, leadership and coordination both internal and external to the state (Goodwin 1998).

Lassa (2010) views disaster governance as the way in which society manages the full array of its disaster risks that may be triggered by geological hazards (such as earthquakes), climate change and hydro-meteorological hazards (such as floods and cyclones), conflict and war. The United Nations Development Programme (UNDP) (2004) cited in Lassa (2010:27) divides disaster-risk governance into three categories. Firstly, *economic governance* refers to decision-making processes that affect a country’s economic activities and the implications thereof for equity, poverty and quality of life. Secondly, *political governance* entails the process of decision making to set legislative processes, formulate laws and write regulations and policies, which is referred to by HFA as the strong institutional basis for implementation. Lastly, *administrative governance* is defined as the system of policy implementation that requires the existence of well-functioning government organisations at national and local levels. They play a role as enforcers of regulations related to disaster mitigation, build code enforcement, plan for land use and environmental risks and monitor human vulnerability and safety standards (see UNDP 2004:1975). The literature is becoming clearer on the elements of disaster governance, including risk assessment, risk management and risk communication, which require understanding of formal and informal institutions, social-economic contexts within which risk is evaluated and the involvement of actors and stakeholders who represent them in political and policy arenas that range from the local to the global level (Fan 2015; Renn 2008; Renn & Walker 2008; Tierney 2012). What is missing from the literature is the critical role of leadership in effective disaster governance.

The literature is clear on the five common elements of the disaster cycle that should be a concern of DRR leadership: prevention, mitigation, preparedness, response and recovery. UNISDR (2009) views prevention as the outright avoidance of the adverse impact of hazards and related disasters. However, as the complete avoidance of losses is not often feasible, the task transforms to that of mitigation, which encompasses engineering techniques and hazard-resistant construction as well as improved environmental policies and public awareness. Preparedness refers to actions and plans taken before the disaster to achieve an orderly transition from response to recovery. Response tends to be associated with actions taken to absorb the impact of the disaster, including actions to save lives and to prevent further damage to property and livelihood. Recovery refers to those actions taken after the initial impact, including those aimed at achieving a return to normality (Kapucu 2008). In other words, DRR leadership is inclusive of all stages of the disaster cycle.

However, leadership is a highly contested term, meaning different things to different people. Hailey (2006:2) defines leadership as ‘… a process whereby an individual influences a group or individuals to achieve a common goal’. Kouzes and Posner (1995:30) describe leadership as ‘… the art of mobilising others to want to struggle for shared aspirations’. Thus, leadership could be viewed as a process of social influence in which one person can enlist the aid and support of others in the accomplishment of a common task. Whilst these two definitions are not necessarily representative of the numerous and sometimes competing notions of leadership, they point out at least three perspectives on leadership. Firstly, some perspectives focus on the traits of successful leaders such as style and approach (Stogdill 1950), for example, charisma to deal with, amongst others, contingencies and challenging situations (Fielder 1967). Secondly, some focus on transformational leaders with the ability to inspire followers during both normal and troubled times, mainly characterised by four Is: idealised influence (more commonly termed charisma), inspirational motivation, intellectual stimulation and individualised consideration (Bass 1985; De Bussy & Paterson 2012; Yunus & Anuar 2012). Thirdly, some go beyond perspectives on the traits of leaders and transformational leadership, which essentially focus on the person of the leader. Horner (1997) views leadership as a process in which a leader is not seen as an individual in charge of followers. Rather, a leader is seen as member of a community of practice where people are united in a common enterprise and share a history, values, beliefs, ways of talking and ways of doing things (Drath & Palus 1994). The leader in these circumstances is akin to what Zolli and Healy (2012:240) call the ‘translational leader’ who is not rooted solely in their formal status but in their informal authority and cultural standing. Thus, the leadership imperative is centred on influence, facilitation, collective empowerment, networking and coordination, not command and control (Zolli & Healy 2012).

The leadership style in disaster management focused on command and control has a long history that is rooted in the military model of emergency preparedness and response. According to Dynes (1994), the assumption of the command-and-control model should be understood in terms of the three Cs. The first ‘C’ assumes that disasters are characterised by ‘chaos’, and the other two ‘Cs’ suggest that the chaos can be eliminated by command and control. The command-and-control formulation, including its mutations such as Incident Management Systems (Perry 2003) and Unified Command Incident Systems (Buck, Trainor & Aguirre 2006), heavily draws from World War II and the Cold-War era. It recognises the capacity of military organisations to deal with disasters, a view which has been deeply embedded in disaster-management and civil-protection organisations (Dynes 1994). However, Carvalho (2015) states that the wide-ranging systemic breakdowns, interdependencies and the uneven effects of catastrophes indicate that current practices of command-and-control leadership are insufficient to the challenges of ‘hyper-complex’ events. Instead, Carvalho (2015) makes the following claim:

… transformative leadership that encourages a sense of empowerment, ownership and engagement within the community is required, incorporating a relational approach that constitutes a nexus between leader and follower, with both sides exercising agency. (p. 82)

In the current study, leadership is viewed as a relational and distributed activity. This view of leadership is consistent with the governance approach to DRR. We posit that leadership is one of the essential elements of DRR governance at global, regional, national and local level. The DRR ‘platforms’ require leadership in order for the stakeholders, comprising United Nations (UN) agencies, government ministries, departments, civil-society groups, donors, International Non-Governmental Organisations (INGOs) and community organisations to share DRR ideas, experiences, expertise, case material and views.

**FIGURE 1:**
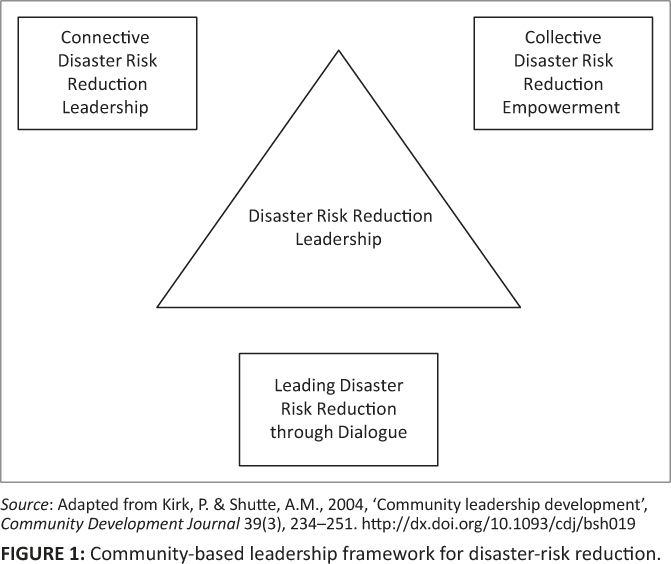
Community-based leadership framework for disaster-risk reduction.

Analysing DRR leadership can be problematic as it requires a framework which captures relational, transformational, translational and distributed leadership, which may also assimilate relevantly to DRR governance. Kirk and Shutte’s (2004) framework is adapted to analyse DRR leadership (Figure 1) as it captures the basic components of relational, transformational, translational and distributed leadership. The framework has three components of community leadership, which are dialogue, connective leadership and collective empowerment.

Dialogue or collective thinking helps communities and their leaders create a climate that can lead to greater collaboration, fluidity, collective and inclusive learning, and sustainability (Kirk & Shutte 2004) of DRR activities. Kirk and Shutte (2004) further contend that dialogue can lead to agreements even if community members may not agree to the direction to be followed. They further state the conditions under which dialogue can yield positive results. In the context of DRR, community members should feel good enough, safe enough and the environment should be open enough to identify and map hazards, express their vulnerability and capacity, and deal with uncertainties and difficulties in order to construct appropriate responses to the realities obtaining in their community. In this way, DRR leadership could be viewed as a collective distributed phenomenon that is constructed around dialogue as a means to enhancing resilience to disasters.

Connective leadership also integrates the gendered notions of leadership (Kirk & Shuttle 2004). Kirk and Shutte (2004) further state three concerns of connective leadership. Firstly, it helps individuals integrate their desires with community or organisational goals. In the context of DRR, an individual identifies DRR goals, takes ownership of the goals and chooses actions to achieve the goals. Secondly, connective leadership encourages members to collectively explore the possibilities and potential of connecting with a common goal. In relation to DRR, this might include team work before and after a disaster, particularly in community projects. Finally, connective leadership helps create and sustain a creative space where collective leadership can flourish. The DRR leadership will foster collaboration and enable different voices to be heard, including those of vulnerable groups such as children, women and the elderly and disabled persons. The leadership will not necessarily come from just one direction but from multiple directions.

Kirk and Shutte (2004) also state that collective empowerment helps individuals to find their place, their role, their identity and their voice in the system. As individuals become interconnected in all parts of the system and have a clear conception of their roles, they develop fruitful relationships with others, clarity about purpose, meaning and value in their work. Consequently, individuals then take responsibility for themselves in relationship to others, their work, the system they are in and the larger environment that contains their system. Kirk and Shutte’s (2004) framework promotes partnership between individuals and groups who essentially need to work together to build resilient communities where the values of empowerment, inclusivity, accountability, collaboration and transparency are paramount.

## Methodology

This study adopted PAR as research method. It tends to be associated with social transformation as it was assumed that it would increase accountability and, where possible, translate into political action (Gibson 2004; Sarantakos 2006) at the local level. Mainly associated with Kurt Lewin’s action research in the 1940s in USA and later with Paulo Freire’s work in Brazil, Mahatma Ghandi’s work in India and Julius Nyerere’s work in Tanzania, PAR can empower the oppressed to transform society and assert their rights (Savin-Baden & Wimpenny 2007). In this way, PAR stand in sharp contrast to the positivist epistemologies of knowledge construction where knowledge is seen as a free-standing unit independent of the researcher (McNiff & Whitehead 2002). In this study, researchers and participants identified the problems and formulated actions together to change the situation for the better.

The study focused on the roles of and links between vulnerable communities, government institutions at district and national level and humanitarian agencies on disaster preparedness and mitigation. It examined how these agencies could be made more responsive to the needs of poor people by adopting a livelihood-centred approach to disaster management. The PAR study worked in partnership with two local NGOs, Hlekweni Friends Rural Service Centre and Organization of Rural Associations for Progress. Other participants in the project included World Vision, Tjinyunyi Babili Trust (a local NGO), Patriots Development Trust, Bulilima Business Development Association and Lutheran Development Services. Government departments, ministries and extension agencies included the Department of Agricultural Technical Extension; Veterinary Services; Environmental Management Authority; Forestry Commission; the Ministries of Health, Youth, Gender and three rural district councils. Other partners included the National University of Science and Technology, Matopos Research Station, International Crops Research Institute for the Semi-Arid Tropics (ICRISAT) and the Provincial Monitoring and Evaluation Unit.

This study mainly used the Participatory Disaster Risk Assessment (PDRA) framework that helped communities in the targeted wards to prioritise hazard risks that needed prevention, transfer or adaption to. Once the lists of hazards were drawn up, Ward Disaster Risk Management Committees were selected by the community members. This did not involve forming new or parallel structures but strengthening existing ones, particularly the conservation committees. The Ward Disaster Risk Management Committees underwent two-day, ward-based, community-training workshops on DRR issues. The training included DRR leadership, conducting PDRAs, climate-change adaptation and environmental management.

However, PAR is not a magic wand for social change. Not only was this project difficult to organise as it required financial, material and human resources, but it also required a longer time frame to realise the envisaged social change. Also, whilst heated debates exposed several weaknesses in leadership, it also raised ethical and security issues in Zimbabwe’s highly politically polarised context which put both the organisers and the participants at risk of being associated with certain political beliefs. To maintain confidentiality, pseudonyms (X, Y and Z) are used for the wards and some organisations.

## A framework for disaster-risk reduction in Zimbabwe

In Zimbabwe, DRR leadership is derived from the 1989 *Civil Protection Act*, which replaced the 1982 *Civil Defence Act*. In theory, the 1989 *Civil Protection Act* marked a shift from the 1982 *Civil Defence Act*, which emphasised the military and authoritarian approaches of the Cold War era (1948–1989), to collaboration and an information-sharing model of disaster management (Alexander 2002). The downside of the civil-defence model’s mainly command-and-control style is that it can become an instrument of repression. Furthermore, it can be used as a strategy for ensuring that protests are repressed and revolts subdued, even when these are stimulated by a desire to defend or restore democratic rights (Alexander 2002). In contrast, whilst some of the 1982 *Civil Defence Act* elements were retained, for example, the definition of ‘civil protection’ and National Civil Protection Fund, the 1989 *Civil Protection Act* broadened and ‘civilianised’ disaster management by extending civil protection to include natural hazards such as floods and droughts.

The 1989 *Civil Protection Act* authorises the establishment of hierarchical DRR institutions in Zimbabwe (Parts IV and V). The Civil Protection Directorate, a department under the Ministry of Local Government and Urban Development, is responsible for the coordination of civil protection in Zimbabwe. At the national level, the National Civil Protection Committee or National Platform is led by the Director of the Department of Civil Protection. At the provincial level, the Provincial Civil Protection Committee is led by the Provincial Administrator, and at the district level, the District Civil Protection Committee is led by the District Administrator. The decentralised structures are accountable to the upper structures and are required to produce emergency preparedness and response plans that are not only supposed to be activated during a disaster but also, in theory, form the basis of the national preparedness and response plan for disasters.

There are at least three structural and functional challenges imposed by the *Civil Protection Act*, which renders it an obsolete piece of DRR legislation in Zimbabwe. Firstly, at the sub-national level, there are neither specifically designated officials nor resources to support the functions of the Civil Protection Directorate. The resources are normally made available when a disaster occurs, through the National Civil Protection Fund. Thus, the functions of the Civil Protection Directorate are at the discretion of the Provincial and District Administrators offices and tend to rely on support from the National Civil Protection Fund and non-government bodies such as UNDP, UNICEF, Office for the Coordination for Humanitarian Affairs (OCHA), Zimbabwe Red Cross Society and Save the Children, Oxfam and World Vision.

Secondly, the 1989 *Civil Protection Act* is not in sync with either the elements of the Hyogo Framework for Action 2005–2015 (HFA) or its successor, the Sendai Framework for Disaster Risk Reduction 2015–2030 (SFDRR). The *Civil Protection Act* barely addresses the optimal achievement of the SFDRR, which is ‘… substantial reduction of disaster risk and losses in lives, livelihoods and health and in the economic, physical, social, cultural and environmental assets of persons, businesses, communities and countries’ (UNISDR 2015:12). Like the HFA, the SFDRR points clearly to the role which the United Nation’s Member States see DRR playing in achieving the parity aspired to by development initiatives, including the proposed Sustainable Development Goals (SDGs) that will replace the current Millennium Development Goals (MDGs). Thus, the 1989 *Civil Protection Act’s* focus on emergency preparedness and response does not adequately address the contemporary disaster-management issues which emphasise proactively and pre-emptively reducing risks by embedding them in a sustainable-development framework and vice versa in an attempt to increase the resilience of both government and communities.

Thirdly, the *Civil Protection Act* lacks any emphasis on the participation of stakeholders and affected communities to build resilience. This is inconsistent with the neo-liberal emphasis on the reduced role of the state (Schuurman 1997) as well as the post-Marxists’ belief in empowering the poor, promoting self-government and enabling local people to relate their stories and livelihoods and to preserve and promote local identity, culture and autonomy (Rodríguez-Pose & Sandall 2008) in enhancing their disaster resilience.

To address the shortcomings of the 1989 *Civil Protection Act*, the Zimbabwe government has produced draft legislative policy frameworks in the form of the Disaster Risk Management Bill of 2011, the Disaster Risk Management Policy of 2011 and the Disaster Risk Management Strategy of 2012. Given the volume of ‘urgent matters’ that the government of Zimbabwe needs to re-align with the 2013 constitution, it is highly unlikely that the disaster legislation would be approved any time soon. Nonetheless, several agencies have been inductively involved in piloting DRR projects to produce evidence of doing DRR in Zimbabwe, which may be used to advocate for the approval of draft legislations.

## The disasters context in Matabeleland South Province

Located in the southern part of Zimbabwe, Matabeleland South Province is one of Zimbabwe’s ten provinces. It covers an area of 54 172 km^2^, which makes up 13.8% of Zimbabwe’s total area. It has a population of 683 893 (ZIMSTAT 2012 Census), accounting for about five percent of Zimbabwe’s population. Matabeleland South Province shares an international boundary with South Africa and Botswana in the south and national boundaries with Matabeleland North and Midlands in the north and Masvingo Province in the east. There are three major urban settlements, namely Gwanda, Plumtree and Beitbridge, and seven rural district councils, namely Beitbridge, Bulilima, Gwanda, Insiza, Mangwe, Matopo and Umzingwane.

Matabeleland South Province lies in Natural Farming Region (NFR) Four and Five, which have the lowest agriculture potential (Table 1). The mean maximum daily temperatures vary from about 22 °C to 26 °C in winter and from about 30 °C to 34 °C in summer. However, in summer, absolute daily maximum temperatures may exceed 40 °C whilst in winter they may fall below 0 °C (Food and Agricultural Organisation [FAO] 2015).

**TABLE 1:** Zimbabwe’s natural farming regions.

Region	Characteristics
One	1500 mm or more rainfall per annum with some rain in all months of the year and relatively low temperatures, specialised and diversified farming.
Two	700–1500 mm rainfall per annum with rainfall confined to summer, suitable for intensive farming.
Three	500–700 mm rainfall per annum with relatively high temperatures, infrequent, heavy showers and subject to seasonal drought, semi-intensive farming region.
Four	450–600 mm rainfall per annum and subject to frequent seasonal drought, semi-intensive farming region.
Five	Less than 500 mm rainfall per annum, erratically distributed, extensive farming region.

The rainy season occurs between November and March with the rest, April to October, being the dry season. Mid-season dry spells occur around January and February. Table 2 shows the mean annual (01 July – 30 June) and seasonal (01 October – 30 April) precipitation by ENSO phase between 1951 and 1991.

**TABLE 2:** Mean annual and seasonal precipitation (mm), 1951–1991.

Type of precipitation	Site						
	Karoi (NFR II)		Gweru (NFR III)		Masvingo (NFR IV)		Beitbridge (NFR V)	
	Annual	Seasonal	Annual	Seasonal	Annual	Seasonal	Annual	Seasonal
La Niña	740	719	750	726	725	692	332	301
Neutral	689	677	680	659	652	610	371	341
El Niño	615	608	619	591	565	526	302	278

Source: Phillips, J.G., Cane, M.A.& Rosenzweig, C., 1998, ‘ENSO, seasonal rainfall patterns and simulated maize yield variability in Zimbabwe’, *Agricultural and Forest Meteorology* 90, 39–50, http://dx.doi.org/10.1016/S0168-1923(97)00095-6

The mean annual and seasonal rainfall for Beitbridge, which lies in Matabeleland South, is below that of Karoi, Gweru and Masvingo, which lie in Natural Regions Two, Three and Four, respectively. The hot and arid conditions make cropping a risky venture, unless under irrigation. The area is suitable for animal husbandry. These conditions make the province vulnerable to high levels of food insecurity. During the drought of 1991 and 1992, when rainfall during the agricultural season averaged 315 mm, Matabeleland South Province was one of the hardest-hit provinces. Although Zimbabwe traditionally has a medium to low risk for disasters, Matabeleland South is a frequent victim of experiencing slow-onset disasters, triggered by weather-related hazards, particularly droughts. The livelihood insecurity in this province is further compounded by the lack of water reservoirs since it is a very low-rainfall area. Consequently, the majority of people in this province have diversified out of agriculture into harvesting forest products for both subsistence and commercial purposes. The area also has occasional episodes of flash floods. Like other provinces of Zimbabwe such as Matabeleland North and Bulawayo, Matabeleland South Province has also been affected negatively by HIV and AIDS. Apart from the natural hydro-meteorological hazards, human-made hazards also affect the three districts (Bongo 2009). The hyperinflationary environment in Zimbabwe, which was more pronounced between 2007 and 2009, impacted negatively on the livelihood and survival strategies of the population in Matabeleland South. Inflation shot up to a high of 500 billion percent. Many people could barely access basic commodities for survival as well as school fees. The economic fabric had largely collapsed.

Nonetheless, the socio-economic situation in Zimbabwe has generally improved, following the formation of the Government of National Unity between the Zimbabwe National African Union – Patriotic Front (ZANU PF) and the Movement for Democratic Change (MDC) parties in 2009 and the subsequent elections in 2013. However, with high unemployment levels of up to 80%, poor social-protection measures, rainfall variability due to climate change and poverty, the situation is unlikely to have improved the lives and livelihoods of people in Matabeleland South Province.

## Case one

### X ward’s disaster-management committee

X is a ward in Bulilima District, Matabeleland South Province, Zimbabwe. X is one of the 12 wards that was targeted for this study. In enhancing community capacity, the first step was to establish X ward’s Disaster Risk Management Committee (the Disaster Committee) that would provide leadership during and beyond the implementation period. The Committee was led by a male chairperson, and it consisted of seven members, one female and six male. These were elected by the community, following the DRR awareness campaigns and meetings between the community and the consortium of partners working within the ward to reduce the risk of disasters. After the committee was elected by the community, committee members went on to select their own office bearers, based on their contribution to past initiatives in community development as well as expertise in management and community mobilisation. The person elected into the office of the chairperson was very influential in the ward. His influence was based on the fact that he had had some exposure at working in a city for some time, and this enabled him to be more knowledgeable in most issues than other people. The chairperson presided over meetings and liaised with staff of the RDC and NGOs working in the area. The vice-chairperson presided over committee meetings in the absence of the chairperson. The secretary organised meetings and took minutes. He was also responsible for record-keeping and communicating vital information concerning disasters to the committee and community at large.

The committee held their meetings at least once every month though there were cases where they had *ad hoc* meetings in the event of an emergency. The committee jointly prepared the agenda. Most agenda items focused on the incidents of danger or lack of safety for people and domestic animals as they attempted to cross the gully during the rainy season. Other issues included measures to reduce the spreading of the gully towards homesteads, the main road, the business centre and the school yards. At times, people who were more knowledgeable in the issues being discussed would chair the meeting. For instance, when discussing the schools in relation to the risks posed by the gully, the local headmaster or one of the teachers representing him would chair. Such incidents, though, were the exception rather than the norm. In keeping with the training they received on participatory approaches to development and DRR, the committee consulted widely with representatives from various villages in the ward before they would pass major decisions. They also sought the advice of the local elite like business people, teachers, school heads and retired civil servants like ex-police personnel.

The Disaster Committee was trained on disaster concepts, risk assessment, disaster prevention and mitigation, preparedness and response as well as search and rescue. The training needs were identified on the basis of the project baseline on DRR, which had been compiled in 2008. These needs included risk assessment, adaptation to climate change, vulnerability and capacity analysis. Some of the needs were also highlighted by community members during initial project-inception meetings and biannual project reviews. Information on capacity gaps was provided by the RDC and various extension agencies from government like AGRITEX, Veterinary, Forestry Commission and the Environmental Management Agency (EMA). Local-community involvement in the Communal Areas Management Programme for Indigenous Resources (CAMPFIRE) projects also shed some light on capacity gaps. Following the training, particularly the participatory vulnerability and capacity assessments conducted between 2008 and 2009, X community decided to give first priority to gully reclamation as a way of preventing the risk of a disaster. The gully had become a cause for concern to X community as it has been growing uncontrolled for years.

As a result, preparatory meetings were conducted, and with support from one of the partner non-governmental organisations, the community engaged an engineer to assist in designing the most appropriate strategies for gully control. The team working with the engineer produced a bill of quantities for the gully rehabilitation, stones, cement and tools. In implementing the recommendations from the engineer, the Disaster Committee hired a tractor, using resources from the much-cited CAMPFIRE. The CAMPFIRE proceeds were for the benefit of the ward at large and not necessarily for the committee. There was recognition on the part of the community that it would be beneficial for the community if the DRR and CAMPFIRE interventions pooled resources, thereby complementing each other, cutting down on costs and duplication. The tractor ferried the boulders from a nearby stream to the gully site. These boulders would be part of the material to be used for gully reclamation. The local community members’ own contribution to the gully-reclamation project, apart from their labour, was the use of their own tools free of charge. Building gabion boxes, planting of *ingotsha* trees and catchment-management campaigns were also considered as additional supporting strategies.

At this stage, another international non-governmental organisation joined the gully-reclamation project. This non-governmental organisation provided food to the people who were working on rehabilitating the gully. The committee did not request the food and neither did it invite the NGO to assist. Rather, when the NGO employees heard that a DRR initiative was taking place in the ward, they indicated to the ward that they intended to assist in the rehabilitation of the gully by providing food for those who worked on the gully project. The food included maize meal, cooking oil, beans and some pulses. NGO staff distributed the food directly to the community recipients. However, this non-governmental organisation managed to provide food to the community members working on the gully for three months only, owing to resource constraints. When the food support was withdrawn, community members were disgruntled. They stopped reclaiming the gully, arguing that they could not work without being provided with food or any other form of payment. For close to 10 months, there was little progress on the gully-reclamation project. Consequently, in 2010, the Ward Disaster Committee decided to shelve the gully-reclamation project. Moreover, the Disaster Committee feared that the material could be misused, stolen or vandalised. As a result, the materials that were procured for the gully-rehabilitation project were diverted to the construction of a classroom block at a secondary school.

## Case two

### The fire incident in y district

Veldt fires have become a cause for concern in Zimbabwe, Y district included. These fires result in loss of lives and livelihood. By end of July, in 2012, 799 fires have been reported to the Environmental Management Agency, including the loss of five lives. Veldt fires can cause extensive damage to the environment, animal deaths, injury, loss of property, air pollution, disruption of power and communication and huge economic losses. Veldt fires can be caused spontaneously or intentionally or unintentionally by people. According to EMA, about 5% of the country, equivalent to 200 585 hectares, has an extremely high risk for fires.

A fire broke out in Y District, one of the districts in Matabeleland South Province, in late 2009. The veldt fire occurred in Y District after most of the district stakeholders were trained on disaster-risk reduction. The training covered the basics of disaster-risk assessment, hazards and their evolution into disasters, CBDRM, search and rescue, participatory capacity and vulnerability assessments and the basics of preparedness-and-response planning. The officers also participated in participatory vulnerability-and-capacity assessments as part of the drafting of their District Disaster Management Plan. According to the *Civil Protection Act* of 1989, the District Administrator (DA) is the coordinator of the District Civil Protection Committee. According to Section 18 of the Act, as an Area Civil Protection Officer (at District level), the DA is mandated to do the following, amongst other things:

- the establishment, maintenance and command of civil-protection organisations
- the provision, operation and coordination of all civil-protection services and activities connected with civil protection
- giving such orders and taking such measures during a state of disaster as in his opinion are reasonably necessary in order to deal with such a state of disaster
- the coordination of the use of materials and services made available by government ministries, local authorities, statutory bodies and other organisations during a state of disaster
- the preparation of reports on civil protection generally in his civil-protection area whenever he is required to do so by the provincial civil-protection officer.

Given such a scenario, disaster issues brought to the attention of the RDC by the ward committee would ideally have been tabled before the DA, who would then take the necessary action. However, owing to a number of reasons, this was not the case. One of the reasons was that, before the project was introduced, most DRR structures and activities were defunct. This was caused by a lack of role clarity and capacity on DRR on the part of the office bearers mandated to lead in this sector.

Upon hearing about the veldt fire, the DA, in the company of two officials, rushed to the scene of the outbreak. The group arrived at the scene of the veldt fire, disembarked from their vehicle and stood at akimbo as the fire spread before their eyes. As the district level, civil-protection leadership, they had at least gone to ‘assess’ the fire outbreak. It was clear that, as the leader, the DA had to be seen to be doing something about the fire, but he actually did not do what was needed. The DA and his team did not find out how the Ward Disaster Management Committee was handling the fire incident. The training had gone on well with people coming out of the training and planning process highly motivated and raring to go. However, this incident glaringly portrayed the lack of empowerment, resources and initiative to deal with the fire.

## Case three

### Z ward’s Participatory Vulnerability and Capacity Assessment process and the food incident

A participatory process of vulnerability-and-capacity assessment was one of the key activities of this study. The usual arrangement for such community processes was that the leading international non-governmental organisation and its partners would contribute a certain percentage of the food and supplies whilst the community at ward level did the same. These contributions were for the meals that were prepared at the sites where the training-and-planning workshops took place for at least three days at a time. Since people who underwent training and did the planning spent the entire day at the planning site (usually a school or community hall), meals would be prepared to feed them, specifically breakfast and lunch. Community contributions to the workshops were important as it was assumed that such contributions would not only reduce the dependency syndrome for donors funding but also enhance ownership of the project by the community members.

Some few weeks before the participatory training on vulnerability-and-capacity assessment was conducted, the supporting international non-governmental organisation’s project staff went to Z ward to deliver money, maize-meal flour and cooking oil for feeding the participants. The money would be used to buy meat, vegetables and refreshments. The items were given to the ward councillor for safe keeping. He was male.

On the first day of training, and by mid-morning, a group of five cooks who had volunteered to prepare lunch for the participants had lit a fire, cleaned their utensils and were ready to start cooking. They waited for the delivery of food items from the councillor. The food could not be delivered. The cooks decided to approach the councillor. When the councillor was asked by the cooks about the whereabouts of the money and the food that he was given, he told them that the money was used for buying refreshments such as biscuits and soft drinks for the participants. The cooks were not satisfied by the councillor’s explanation. They then approached the other village-committee members and village heads who demanded an explanation from the councillor about the cash and food stuff that were delivered to him by the project staff. In response, the councillor produced receipts indicating that he had bought biscuits and cooking oil at inflated prices.

The argument attracted the attention of participants, who joined the cooks, village-committee members and village heads to reject the councillor’s fake receipts. The group did not want to take the matter to the police for the councillor to be arrested. They felt that they would lose out in such a process. It was better to use local means of solving their problems as they knew each other very well. They requested the councillor to find means of feeding the participants. As a result, the councillor was forced to slaughter his own chickens so that he could provide nourishment for the trainees. At the end, the councillor was embarrassed. There were strong sentiments from the participants that they would vote him out during election time.

## Discussion

The three case studies, X, Y and Z, reveal various inherent tensions which the DRR leadership experienced following the capacity building by the PAR project (Table 3). The tensions are mainly around the shift from government-type DRR leadership to governance-informed leadership. X case study reveals some elements of distributed leadership which tends to be associated with a governance ‘mentality’. In contrast, case-study Y reveals a disconnect between government and local leadership. Whilst case study Z has similarities with case study X, Z appears to emphasise accountability and conflict resolution. These issues are discussed below.

**TABLE 3:** Summary of leadership styles in Ward X, Y and Z.

Ward	Leadership style		
	Person-centred leadership	Command and control leadership	Distributed leadership
X	-	-	✓
Y	-	✓	-
Z	✓	-	✓

With the exception of Y, the other two case studies, X and Z, illustrate a gradual shift from government as well as person-centred leadership to governance-based leadership. Case Y reflects the top-down, command-and-control based leadership to disaster response, reflecting the civil-defence approach of the Cold War period (Alexander 2002) of dealing with chaos through command and control (Dynes 1994). The DA, who leads the District Civil Protection Committee, appeared to be disconnected from the Ward Disaster Management (DRM) Committee. Instead of establishing the Ward Disaster Management’s response plans, available resources and external resources needed to contain the fire, the administrator watched helplessly as the fire destroyed vegetation and livelihood. Whilst Y may not only be an extreme case that is not necessarily representative of Zimbabwe’s districts, there are several questions that need further research. What happened to the coordination and facilitation role of the DA, the DRR leader, for example? Even from a civil-defence vantage point, what preparedness and response plans existed, if any at all? This suggests that, even after capacity-building interventions, there appears to be no magic wand to institute a ‘quick’ shift from a civil-defence to an agency-based resilience-building paradigm. The reactive top-down, command-and-control leadership style, where the affected local communities are viewed as vulnerable and with little if any ability to respond to an emergency, is likely to continue surfacing. The ‘government’, response-based leadership style tends to bring quick wins and results which can help spruce, in some cases, waning political image and support from the general public.

However, in case X, the leader appeared to facilitate dialogue amongst members of the ward’s DRM committee. This has helped members to make joint and binding decisions. For example, the ward’s DRM Committee was involved in the selection of the civil engineer who provided technical support on the gully reclamation. The ward’s DRM Committee also managed to mobilise local resources to hire a tractor, using CAMPFIRE project funds. This indicates that the leadership was to some extent underpinned by elements that are consistent with translational leadership, including but not limited to dialogue, influence, facilitation, collective empowerment, networking and coordination (Carvalho 2015; Kirk & Shuttle 2004; Zolli & Healy 2012). Thus, the leader in case X seems to be rooted in the community history, culture and beliefs, which, in our view, can be a galvanising factor for building resilience.

Nonetheless, considering food insecurity in the Matabeleland South Province, food transfer was to a large degree necessary to reduce malnutrition, a disease in its own right, which can predispose children and adults to infectious diseases (Collins 1998). Challenges started to emerge when food support was withdrawn. These challenges could be related to at least two underlying but well-known problems in development theory, policy and practice. Whilst the problems appear to be external to the ward’s DRM Committee, they had a profound effect on the leadership. Firstly, as Matabeleland South Province is a frequent candidate for humanitarian aid, communities have developed a dependency syndrome as they seem to be accustomed to receiving some form of payment for public works, particularly through the food-for-work projects which became popular following 1992’s drought-induced disaster. When the food support was withdrawn, community members declined to work on the gully-reclamation project. Secondly, it appears that there was lack of a contractual agreement on the roles and responsibilities of the community and the supporting NGOs. The question is: Who owns DRR? Was gully reclamation a priority for the community? If not, whose priority was it – that of the NGOs and officials? There are no easy answers to these questions. Rather, these questions remind us to reflect on some of the questions that have been asked by development scholars, including one of Robert Chambers’ most cited questions: ‘Whose reality counts?’ (Chambers 1997). Reflecting on such questions might be useful in not only ensuring the collective empowerment of the leadership. More importantly, reflecting on such questions may remind us to ensure that DRR interventions are rooted in the local community’s realities and institutions which are shaped by, amongst others, shared history, culture, values, beliefs and language through which resilience is passed from generation to generation.

The three case studies still exhibit the legacy of civil-defence command and control. The leader in case study Y appears to be more accountable to the upper echelons of the structure than to local communities. However, why is accountability viewed narrowly whilst it is one of the ‘good’ governance principles? The main reason is that the 1989 *Civil Protection Act* does not mandate civil-protection structure to account downwards to local communities during both pre-and-post-disaster periods. This is contrary to what Rogers (2005) calls ‘real accountability’, which is characterised by upward and outward accountability and open room for manoeuvre to respond to emerging needs.

However, case study Z embodies some aspects of accountability. The cooks, the leaders and the workshop participants demanded that the councillor accounts for the food and money he was advanced by the NGO to cater for their food needs. Even when the participants realised that the councillor had clearly faked the receipts, they opted not to take legal action as that would disrupt their training. Rather, they decided to settle the problem using ‘*ubuntu*’ or ‘*hunhu*’ (people) or traditional ways of conflict resolution. Here people sit down together and dialogue to solve the problem without necessarily taking legal action, which may strain relationships and community cohesion. As a result, the councillor replaced the food and money with his own chickens, which were slaughtered to feed the trainees. What comes out strongly is that the participants warned the councillor that they would vote him out at the next election. As promised, during the 2013 elections, the councillor for ward Z was voted out. This resonates with Aldrich’s (2012) emphasis that communities who actively turn out to vote during elections exhibit high social capital, which both serves as informal insurance and mutual assistance for disaster survivors, thus an indicator of communities resilient to disasters.

## Conclusion

This article has demonstrated that leadership is an indispensable component of DRR governance. Whilst addressing the well-known problems of the reactive response to disaster-risk reduction is by no means less important, it is also vital to integrate local, traditional approaches to ensure the sustainability of approaches to and benefits of disaster-risk reduction. As this study demonstrates, ‘good’ approaches to disaster governance should be underpinned by DRR-leadership frameworks, which should consistently emphasise distributed leadership and power, ownership, coordination, partnership and accountability. More importantly, embedding the approaches should integrate local cultural beliefs that have been passed from generations. Also, DRR should be viewed as a development approach rather than a stand-alone project. Viewing DRR from this vantage point allows applying lessons learned from sustainable-development theory, policy and practice, particularly as it relates to, amongst others, good governance, community-based activities and communities of practice. This study also reveals gaps in disaster-leadership research and scholarship, which apparently has been implied under the DRR-governance rubric. Finally, whilst the findings of this study may be specific to the Zimbabwean context, they may resonate with similar contexts elsewhere.
